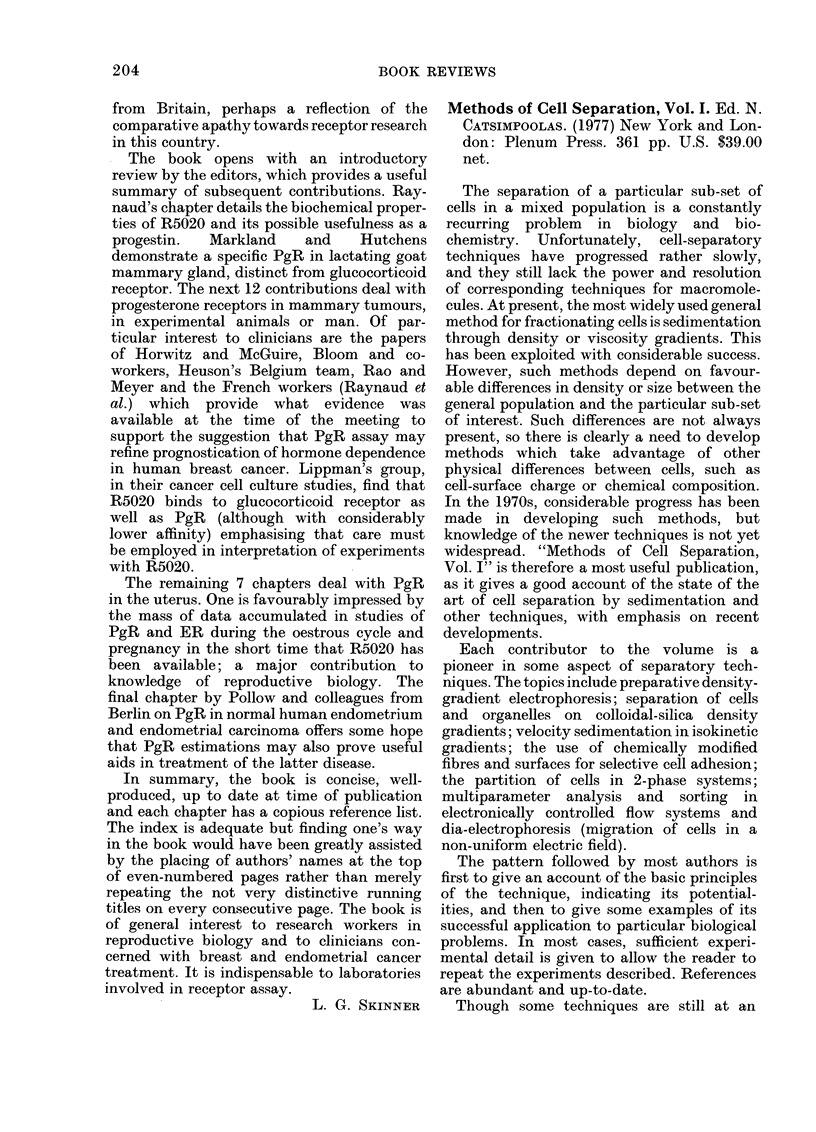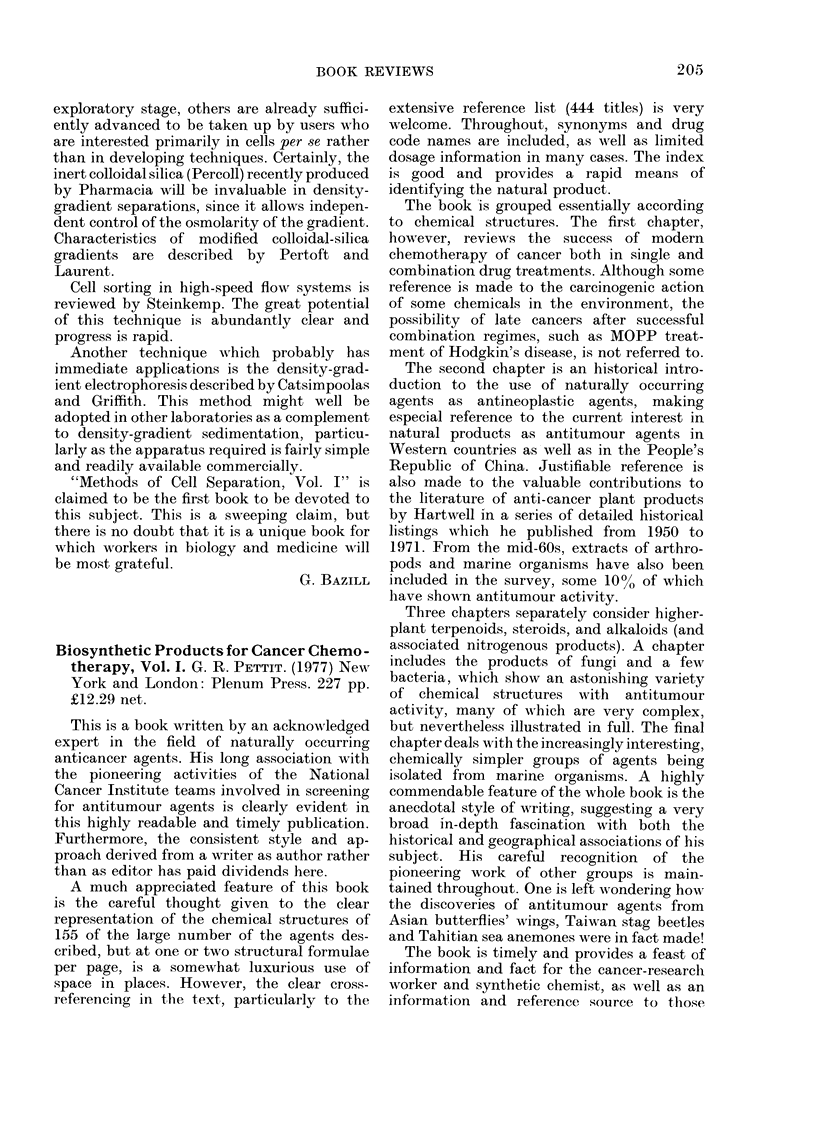# Methods of Cell Separation, Vol. I

**Published:** 1978-07

**Authors:** G. Bazill


**Methods of Cell Separation, Vol. I.** Ed. N. CATSIMPOOLAS. (1977) New York and London: Plenum Press. 361 pp. U.S. $39.00 net.

The separation of a particular sub-set of cells in a mixed population is a constantly recurring problem in biology and biochemistry. Unfortunately, cell-separatory techniques have progressed rather slowly, and they still lack the power and resolution of corresponding techniques for macromolecules. At present, the most widely used general method for fractionating cells is sedimentation through density or viscosity gradients. This has been exploited with considerable success. However, such methods depend on favourable differences in density or size between the general population and the particular sub-set of interest. Such differences are not always present, so there is clearly a need to develop methods which take advantage of other physical differences between cells, such as cell-surface charge or chemical composition. In the 1970s, considerable progress has been made in developing such methods, but knowledge of the newer techniques is not yet widespread. "Methods of Cell Separation, Vol. I" is therefore a most useful publication, as it gives a good account of the state of the art of cell separation by sedimentation and other techniques, with emphasis on recent developments.

Each contributor to the volume is a pioneer in some aspect of separatory techniques. The topics include preparative density-gradient electrophoresis; separation of cells and organelles on colloidal-silica density gradients; velocity sedimentation in isokinetic gradients; the use of chemically modified fibres and surfaces for selective cell adhesion; the partition of cells in 2-phase systems; multiparameter analysis and sorting in electronically controlled flow systems and dia-electrophoresis (migration of cells in a non-uniform electric field).

The pattern followed by most authors is first to give an account of the basic principles of the technique, indicating its potentialities, and then to give some examples of its successful application to particular biological problems. In most cases, sufficient experimental detail is given to allow the reader to repeat the experiments described. References are abundant and up-to-date.

Though some techniques are still at an exploratory stage, others are already sufficiently advanced to be taken up by users who are interested primarily in cells *per se* rather than in developing techniques. Certainly, the inert colloidal silica (Percoll) recently produced by Pharmacia will be invaluable in density-gradient separations, since it allows independent control of the osmolarity of the gradient. Characteristics of modified colloidal-silica gradients are described by Pertoft and Laurent.

Cell sorting in high-speed flow systems is reviewed by Steinkemp. The great potential of this technique is abundantly clear and progress is rapid.

Another technique which probably has immediate applications is the density-gradient electrophoresis described by Catsimpoolas and Griffith. This method might well be adopted in other laboratories as a complement to density-gradient sedimentation, particularly as the apparatus required is fairly simple and readily available commercially.

"Methods of Cell Separation, Vol. I" is claimed to be the first book to be devoted to this subject. This is a sweeping claim, but there is no doubt that it is a unique book for which workers in biology and medicine will be most grateful.

G. Bazill